# Pathophysiology, Functional Assessment and Prognostic Implications of Nutritional Disorders in Systemic Amyloidosis

**DOI:** 10.3390/jcm12020528

**Published:** 2023-01-09

**Authors:** Francesca Dongiglio, Emanuele Monda, Giuseppe Palmiero, Federica Verrillo, Marta Rubino, Gaetano Diana, Annapaola Cirillo, Adelaide Fusco, Erica Vetrano, Michele Lioncino, Martina Caiazza, Giuseppe Cerciello, Laura Capodicasa, Flavia Chiosi, Vincenzo Simonelli, Maria Luisa De Rimini, Francesco Natale, Alessandro Di Santo, Elisabetta Moscarella, Paolo Calabrò, Giuseppe Limongelli

**Affiliations:** 1 Inherited and Rare Cardiovascular Diseases, Department of Translational Medical Sciences, University of Campania “Luigi Vanvitelli”, Monaldi Hospital, 80131 Naples, Italy; 2 Haematology Unit, Department of Clinical Medicine and Surgery, University of Naples “Federico II”, Via S. Pansini, 80131 Naples, Italy; 3 Department of Nephrology, Monaldi Hospital, Via L. Bianchi, 80131 Naples, Italy; 4 Department of Ophthalmology, Monaldi Hospital, Via L. Bianchi, 80131 Naples, Italy; 5 Department of Neurology, Monaldi Hospital, Via L. Bianchi, 80131 Naples, Italy; 6 Department of Nuclear Medicine, Monaldi Hospital, Via L. Bianchi, 80131 Naples, Italy; 7 NeMO-Napoli Clinical Center for Neuromuscular Diseases, Monaldi Hospital, Via L. Bianchi, 80131 Naples, Italy; 8 Institute of Cardiovascular Sciences, University College of London and St. Bartholomew’s Hospital, London WC1E 6DD, UK

**Keywords:** amyloidosis, gastrointestinal disorders, prognosis, nutritional assessment

## Abstract

Gastrointestinal involvement is a common clinical feature of patients with systemic amyloidosis. This condition is responsible for invalidating gastrointestinal symptoms, a significant macro and micronutrient deficit, and is a marker of disease severity. Gastrointestinal involvement should be actively sought in patients with systemic amyloidosis, while its diagnosis is challenging in patients with isolated gastrointestinal symptoms. The nutritional status in systemic amyloidosis plays an essential role in the clinical course and is considered a significant prognostic factor. However, the definition of nutritional status is still challenging due to the lack of internationally accepted thresholds for anthropometric and biochemical variables, especially in specific populations such as those with systemic amyloidosis. This review aims to elucidate the fundamental steps for nutritional assessment by using clinical and instrumental tools for better prognostic stratification and patient management regarding quality of life and outcomes.

## 1. Introduction

Amyloidosis is a pathological condition characterized by the extracellular deposition of fibrils in tissues and organs that form as a result of misfolded proteins [1] The amyloid deposition can affect a single organ (e.g., isolated cardiac amyloidosis) or can be systemic, leading to multi-organ dysfunction and premature death [1].

About 30 different proteins have been found to cause amyloidosis, and the classification of amyloidosis is based on to the precursor protein. In particular, the nomenclature is extremely simple: the letter “A” stands for amyloid; “X” indicates the precursor protein [2]. Theoretically, any protein can be a precursor to amyloid fibrils due to abnormal misfolding.

The most common types of amyloidosis are immunoglobulin light chain (AL) amyloidosis and transthyretin (ATTR) amyloidosis [3]. In AL amyloidosis, previously defined as primary systemic amyloidosis, the precursor protein derives from a clonal plasma cell. In particular, the excessive production of immunoglobulin proteins leads to their misfolding and dissociation of light and heavy chains, with subsequent amyloid fibril formation and infiltration [2,3]. On the other hand, in ATTR amyloidosis, the precursor protein derives from transthyretin produced by the liver [4]. In addition, ATTR amyloidosis is further classified as either wild-type (wtATTR) or hereditary ATTR (hATTR). In patients with wtATTR, the pathophysiology of abnormally misfolding proteins appears to be multifactorial (e.g., ageing factors, chaperon protein alterations) [5]. In contrast, patients with hATTR carry a pathogenic mutation in TTR gene, leading to a less stable transthyretin protein and promoting misfolding and amyloid fibril formation [5].

The clinical presentation and organ involvement in hATTR amyloidosis can be variable. Patients affected can have a prevalent neurological, cardiac, or mixed phenotype according to the type of TTR gene mutation [6]. Those with both neurological and cardiac involvement seem to have a worse outcome [6].

In patients with amyloidosis, cardiac involvement is the most important determinant of adverse outcome [7], and the most associated cardiac phenotype is hypertrophic cardiomyopathy [8,9,10].

In patients with systemic amyloidosis, it is common to observe gastrointestinal (GI) involvement [11]. In particular, the infiltration pattern varies according to the type of amyloidosis. From a general point of view, GI manifestations are caused by the amyloid fibril deposition in the wall of the GI tract (mainly the duodenum, stomach, esophagus, and colorectum) [12]. In addition, amyloid deposition in the hepatic parenchyma can be observed.

**Figure 1:**
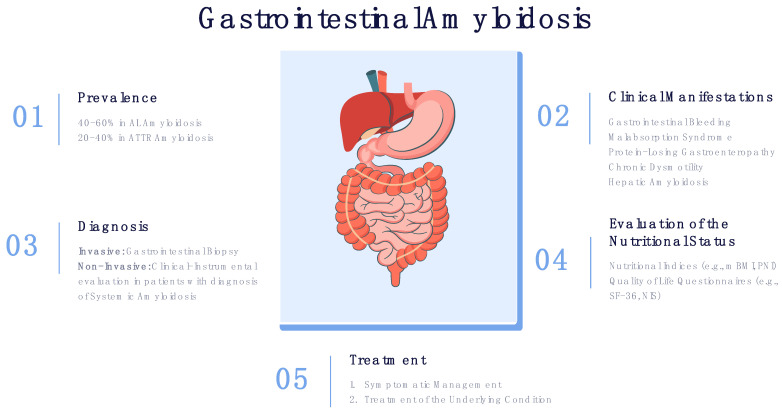
Prevalence, clinical manifestations, diagnosis and treatment of GI amyloidosis.

GI involvement significantly affects the nutritional status of patients with systemic amyloidosis, in some cases leading to severe malnutrition. Consequently, malnutrition leads to a deterioration in the quality of life and overall survival. Thus, clinical manifestations associated with GI should be actively evaluated in all patients with systemic amyloidosis (Figure 1).

This review aims to provide an overview of nutritional disorders’ pathophysiology and prognostic implications in systemic amyloidosis. 

## 2. Pathophysiology of Gastrointestinal Involvement

GI involvement is common in patients with systemic amyloidosis. Its prevalence, severity degree and associated manifestations significantly vary according to the type of amyloidosis (Table 1). GI manifestations depend on the amount and location of the amyloid fibril deposition [13,14]. The involvement of the GI tract is complex and multifactorial.

First, GI manifestations are associated with significant GI amyloid fibril deposition. The GI layers affected are represented by the mucosa and neuromuscular layer. In particular, the duodenum is the most common site of infiltration, followed by the stomach, colorectum and esophagus [12]. Some differences can be observed according to the type of systemic amyloidosis. For example, in AL amyloidosis, it is common to observe the muscolaris mucosa infiltration (leading to mucosal protrusions), while in AA amyloidosis, the mucosa can be significantly affected, leading to erosions and friability [15]. In addition, amyloid infiltration can involve the submucosal plexus and the myenteric plexus, leading to abnormalities in motor, sensory and secretory functions [16].

Second, several mechanisms contribute to GI manifestations in patients with significant cardiac involvement and congestive heart failure. The two main factors responsible for GI involvement are bowel edema and hypoperfusion [17]. Patients with heart failure exhibit increased sympathetic activity, responsible for blood redistribution with significant flow reduction to the GI tract. This flow reduction leads to intestinal ischemia and increased intestinal mucosa permeability [18]. Thus, endotoxins may enter the systemic circulation with subsequent monocyte and macrophage activation and pro-inflammatory mediator releases, leading to inflammatory status. In addition, the diminished GI circulation may contribute to local edema of the bowel wall (mainly caused by volume overload observed in patients with heart failure) and to malabsorption and barrier dysfunction of the mucosa [18].

Third, autonomic dysfunction, mainly observed in patients with hATTR amyloidosis, is a significant determinant of GI manifestations. Autonomic symptoms are present in 50% to 80% of patients with hATTR amyloidosis and may appear at diagnosis or later during the lifetime course [19]. Different mutations carry different risks for autonomic dysfunction. All segments of the GI tract could be involved, contributing to high inter-individual variability in clinical presentation and symptom fluctuation [19]. Clinical manifestations induced by autonomic dysfunction are mainly due to dysmotility disturbances [20].

## 3. Gastrointestinal Manifestations

Patients with systemic amyloidosis can manifest a broad spectrum of GI manifestations. Among the possible clinical manifestations, the most common symptoms described are unintentional weight loss, diarrhea, constipation, and GI bleeding. 

In a retrospective cohort of 583 patients with AL amyloidosis evaluated at a tertiary referral center, the prevalence of GI manifestations was 17%, with abdominal pain, nausea, or vomiting responsible for half of the cases [21]. However, only 45% of symptomatic patients exhibited biopsy-proven GI amyloid [21].

The prevalence of GI symptoms is significantly more frequent among patients with ATTR amyloidosis, especially those with hATTR. Their prevalence was recently evaluated by the Transthyretin Amyloidosis Outcomes Survey (THAOS), a global, multicenter, longitudinal, observational survey designed to understand and follow the progression of ATTR amyloidosis [13,22,23]. The survey analyzed data from 1579 patients with hATTR and 160 patients with wtATTR and described that 63% and 15% reported GI symptoms, respectively, with unintentional weight loss and early satiety as the most common [13]. In patients with hATTR amyloidosis, it was observed that GI symptoms were more prevalent in those with V30M (69%) compared with non-V30M patients (56%). In addition, patients with disease presentation <50 years were more commonly symptomatic than those with later-onset disease presentation. 

GI manifestations are highly variable and reflect the complex pathophysiological mechanisms of GI involvement. Thus, according to the type of systemic amyloidosis and the predominant mechanism of GI involvement, the clinical manifestation can be classified into signs and symptoms caused by GI bleeding, malabsorption syndrome, protein-losing gastroenteropathy or GI dysmotility. In addition, hepatic involvement may occur.

GI bleeding is commonly observed in amyloidosis due to direct vascular and tissue amyloid fibril infiltration, responsible for increased friability and erosions [15,16]. In addition, acquired coagulation abnormalities contribute to increased bleeding diathesis. The most common abnormalities include prolonged thrombin and prothrombin times and decreased factor X activity, associated with hepatic involvement and malabsorption or decreased vitamin K intake [24]. The most common clinical manifestation of GI bleeding is chronic iron deficiency anemia, which results from reduced iron absorption, increased elimination (with GI bleeding) and increased demand [25]. Clinical manifestations associated with acute upper (e.g., hematemesis, melena) or lower GI bleeding are rare [25].

Malabsorption has been observed in nearly 5% of patients with AL amyloidosis [26]. The underlying mechanisms include autonomic neuropathy, amyloid fibril infiltration of the mucosa, ischemia and bacterial overgrowth related to dysmotility [27]. Symptoms vary according to the areas of the bowel involved and are usually progressive. In some cases, the severe GI involvement may lead to protein-losing enteropathy, a condition characterized by an excessive loss of proteins through the GI tract and responsible for hypoproteinemia [27].

GI dysmotility due to autonomic neuropathy and infiltration of the GI autonomic system is responsible for a large part of clinical GI manifestations. Patients may present with nausea and/or vomiting, constipation, alternation of diarrhea and constipation, fecal incontinence, or clinical features of chronic intestinal pseudo-obstruction [13].

Thus, patients should be regularly evaluated for warning signs (i.e., weight loss; early satiety, nausea and vomiting; constipation, an alternation of diarrhoea/constipation, diarrhoea and faecal incontinence) for early referral and intervention [13].

Endoscopic biopsy with the histological demonstration of amyloid deposition in the GI tract represents the gold standard for the diagnosis of GI amyloidosis. The degree and the rate of endoscopic findings vary among the GI tract [28]. Although the frequency of amyloid deposition varies according to the type of amyloidosis, the diagnostic rate is higher (up to 100%) when biopsies are performed in the duodenum compared with those performed in the stomach, colorectum or esophagus [28].

## 4. Malnutrition and Evaluation of the Nutritional Status

Malnutrition is a major determinant of survival and quality of life in patients with systemic amyloidosis [29,30]. Caccialanza et al. analyzed the anthropometric, biochemical and clinical parameters of 106 consecutive patients with histologically proven AL amyloidosis [29] and observed that malnutrition was common in these patients. They found that unintentional weight loss (with a median weight reduction of 11%) was present in 55% of patients and that the body mass index (BMI) was lower than 22 kg/m^2^ in nearly one-quarter of patients. The amount of weight reduction is significantly greater in those with cardiac involvement. [29]. Similarly, another study aiming to assess the association between nutritional status and quality of life in patients with AL amyloidosis showed that malnutrition was evidenced in about 65% of patients, with a negative impact on quality of life and survival [30].

The pathophysiology of malnutrition is mainly caused by GI and cardiac involvement. As mentioned above, GI involvement mainly consists of autonomic dysfunction, malabsorption and dysmotility [12,13,14,15,16,17,18,19,20]. These processes are responsible for an imbalance between the supply and demand of nutrients, leading, in severe cases, to malnutrition and cachexia.

Cachexia is a complex clinical syndrome characterized by body composition abnormalities (i.e., reduction in muscle mass and peripheral oedema) and progressive weight loss (protein-calorie malnutrition). The reduction of muscle mass leads to sarcopenia and a progressive reduction in quality of life. The sarcopenic patient exhibits difficulty walking with consequent risks of falls and fractures, impoverishing the quality of life up to actual disability or possible premature death.

The evaluation of the nutritional status is based on the following:

- anamnestic and clinical data;
- laboratory parameters;
- assessment of body composition by anthropometry, plicometry and impedentiometry;
- use of nutritional indices (e.g., body mass index [BMI], modified BMI [mBMI], prognostic nutritional index [PNI], nutritional risk index (NRI);

However, the definition of nutritional status is still challenging due to the lack of standardized, accepted and shared international parameters. This challenge is even greater in rare diseases, such as in patients with cardiac amyloidosis (Table 2).

### 4.1. Nutritional Indices

In recent years, several indices of nutritional status have been suggested (Table 2). However, knowledge about their clinical application in patients with systemic amyloidosis is limited.

A large study suggested that according to BMI values, the prevalence of malnutrition ranged from 3% to 42% [31]. However, if used alone, BMI is a poor indicator of malnutrition and misclassifies malnourished patients within the normal range. In addition, it may not be reliable in the presence of confounding factors, such as in the case of fluid overload.

The mBMI, calculated as the product of BMI and serum albumin, is an accurate method for assessing the nutritional status of patients with volume overload [32]. The mBMI is used to overcome some limitations related to the isolated use of BMI or albumin. In particular, the BMI does not consider fluid balance. On the other hand, serum albumin does not provide information on the physical status.

In a recent study [33], the prognostic role of different nutritional indices was evaluated in 50 patients with cardiac amyloidosis (26 with AL and 24 with ATTR). Patients with AL amyloidosis exhibited lower mBMI values, while no significant differences were observed for other nutritional indices. In addition, a low BMI was associated with worse survival, and mBMI emerged as an independent predictor of cardiovascular death [33]. Similarly, low mBMI values have been associated with a worse prognosis in patients with renal AA amyloidosis [34]. Driggin et al. [34], investigated differences in survival among patients with ATTR CA by nutritional status defined by mBMI and serum uric acid. They found that patients with low mBMI and/or low serum uric acid showed reduced survival, but only the latter was an independent predictor of death in their cohort [35].

Other nutritional indices have been proposed for the evaluation of nutritional status (e.g., prognostic nutritional index, nutritional risk index, geriatric nutritional risk index) [36,37,38]. However, their role in patients with amyloidosis has not been explored.

### 4.2. Body Composition Assessment

The independent association between malnutrition, prognosis and quality of life in patients with amyloidosis has been explored in different studies [29,30,31]. The assessment of body composition using outpatient methods allows for monitoring the development of the disease and the nutritional status [29]. Impedentiometry is a method that has acquired considerable importance in evaluating patients suffering from amyloidosis, as it allows the evaluation of the distribution of body fluids, which is useful in patients with significant cardiac involvement and heart failure [39]. 

Bioimpedance Vector Analysis (BIVA) has recently been proposed to detect fluid overload and sarcopenia in AL amyloidosis [40]. It is a non-invasive method that allows the analysis of the body composition in a few seconds thanks to the detection of the impedance, or the “resistance” opposed by the body to the passage of an alternating electric current of very low intensity (400 µÅ) and high frequency (50 kHz). It is a safe, fast and reproducible method that can be easily integrated into clinical practice to detect patients with congestion (even subclinical) and monitor disease progression and decongestion with diuretic therapy [41].

With this method, the two main bioelectrical parameters (whole-body resistance [R] and reactance [Xc] derived from a phase-sensitive 50 kHz signal) are used to describe the hydration state, mainly through the PhA phase angle (the primary BIVA-derived output), which can be considered an excellent indicator of physical state and cellular integrity [42,43]. The phase angle has shown a good correlation with the state of cellular health. Thus, the PhA is a widely used indicator to evaluate the nutritional status and for its prognostic value (mortality, disease progression, incidence of postoperative complications, length of hospital stay). However, it is influenced by the state of hydration, which should be considered when interpreting the measurement of the parameter [43].

Rezk et al. [40] recently demonstrated that bioelectrical impedance (BIA) allows for recognizing changes in body composition in AL amyloidosis. They found that nearly 95% of AL patients had significant fluid retention using BIA technology. In particular, they evaluated fluid overload by estimating the ratio of extracellular water (ECW)/to total body water (TBW) (ECW/TBW). They found that excess extracellular water at baseline predicted survival and was associated with failure to achieve a treatment response [40].

There is currently no data in the literature for using BIA for evaluating patients with ATTR amyloidosis. However, since AL and ATTR amyloidosis are commonly associated with cardiac involvement, leading to congestive heart failure, their use in also managing patients suffering from ATTR amyloidosis is reasonable.

## 5. Conclusions

Systemic amyloidosis consists of a series of diseases characterized by the deposition of fibrillar proteins within organs. The pattern of multiorgan involvement and their dysfunction vary substantially, not only between different types of amyloid but also internally for each type. The diagnostic delay is often due to the difficulty of confirming the diagnosis pathologically, resulting in considerable delays in the diagnosis. Significant progress has been made in our understanding of the pathological physiology of amyloidosis, accompanied by developments in treatment that result in improved organ function, quality of life and patient survival. Gastrointestinal symptoms and malnutrition are common in patients with systemic amyloidosis. They are often multifactorial in aetiology and adversely affect patients’ quality of life and overall survival. These characteristics should, therefore, be actively sought and addressed in all subjects with systemic amyloidosis.

## Figures and Tables

**Table 1:** Gastrointestinal manifestations according to the type of systemic amyloidosis. Abbreviations: GI, gastrointestinal.

Type of Amyloidosis	Gastrointestinal Manifestations	Symptom Frequency
AL (amyloid derived from immunoglobulin light chain)	Diarrhea, weight loss, steatorrhea, anorexia gastrointestinal bleeding, heartburn	Weight loss (45%) Gastrointestinal bleeding (36%) Heartburn (33%)
ATTR (amyloid derived from transthyretin)	Unintentional weight loss, upper GI symptoms as early satiety, nausea and vomiting, lower GI symptoms as constipation, alternating diarrhea/constipation, diarrhea, and fecal incontinence	Weight loss (28%) Early Satiety (25%) Diarrhea/Constipation (23%) Fecal incontinence (6%)

**Table 2:** Definition of the main nutritional indices used in clinical practice.

Nutritional Index	Description	Formula
Body mass index (BMI)	Estimate the total body fat	BMI was calculated as follows: weight/height^2^
Modified BMI (mBMI)	Accurately measures the nutritional status of patients with volume overload	mBMI was calculated by multiplying: BMI × serum albumin
Prognostic nutritional index (PNI)	PNI was independently associated with long-term survival in patients hospitalized for acute heart failure with reduced or preserved ejection fraction	PNI was calculated as follows: PNI = 10 × serum albumin (g/dL) + 0.005 × total lymphocytes (count per mm^3^). Patients with a PNI > 38 are considered normal, those with a PNI of 35–38 are at moderate risk of malnutrition those with a PNI < 35 are at severe risk
The nutritional risk index (NRI)	A relative risk index that allows its classification patients based on the risk of morbidity and mortality	NRI was calculated as follows: (1.519 × serum albumin (g/L) + 41.7 × (present weight/usual weight). The patients with an NRI score of >100 were placed in the no risk group, 97.5–100 mild risk, 83.5–97.5 moderate risk, <83.5 severe risk.
Geriatric Nutritional Risk Index (GNRI)	A relative risk index that allows its classification patients based on the risk of morbidity and mortality	GNRI was calculated as follows: (14.89 × albumin concentration [g/dL]) + (41.7 × [actual bodyweight/ideal bodyweight]). A GNRI < 92 was defined as moderate or severe malnutrition risk, while a GNRI ≧ 92 was defined as low or no malnutrition risk

## Data Availability

No new data were created or analyzed in this study. Data sharing is not applicable to this article.
